# Proximitized Topological Insulator Charge Island Fabricated via *In Situ* Multi-Angle Stencil Lithography

**DOI:** 10.1021/acs.nanolett.6c02350

**Published:** 2026-10-05

**Authors:** Benedikt Frohn, Tobias Schmitt, Vanessa Serrano, Anne Schmidt, Michael Schleenvoigt, Albert Hertel, Benjamin Bennemann, Abdur Rehman Jalil, Detlev Grützmacher, Peter Schüffelgen

**Affiliations:** PGI-9, 28334 Forschungszentrum Jülich and JARA Jülich-Aachen Research Alliance, 52425 Jülich, Germany

**Keywords:** Topological Insulator, Coulomb Blockade, Stencil Lithography, Proximity-Induced Superconductivity

## Abstract

Hybrid superconductor–topological insulator (TI) nanostructures constitute a promising material platform for exploring proximity-induced superconductivity in systems with topologically protected surface states. A key obstacle has been the realization of clean and well-controlled superconductor–TI interfaces, as TI surfaces rapidly degrade under ambient conditions. Here, we introduce a fully *in situ*, multi-angle stencil lithography technique that enables the fabrication of proximitized charge islands in TIs. The approach combines selective area growth of (Bi,Sb)_2_Te_3_ nanoribbons with angle-controlled deposition of diffusion barriers, superconducting Al, and ultrathin oxide tunnel barriers, allowing scalable fabrication of hybrid nanostructures without post-growth processing. Low-temperature transport measurements reveal robust Coulomb blockade and a pronounced suppression of low-energy conductance, which vanishes with a magnetic field, consistent with proximity-induced superconductivity in the island. These results establish a versatile nanofabrication platform that enables access to previously unexplored TI-based hybrid quantum devices and opens new routes for investigating superconductivity in topological nanostructures.

## Graphical abstract

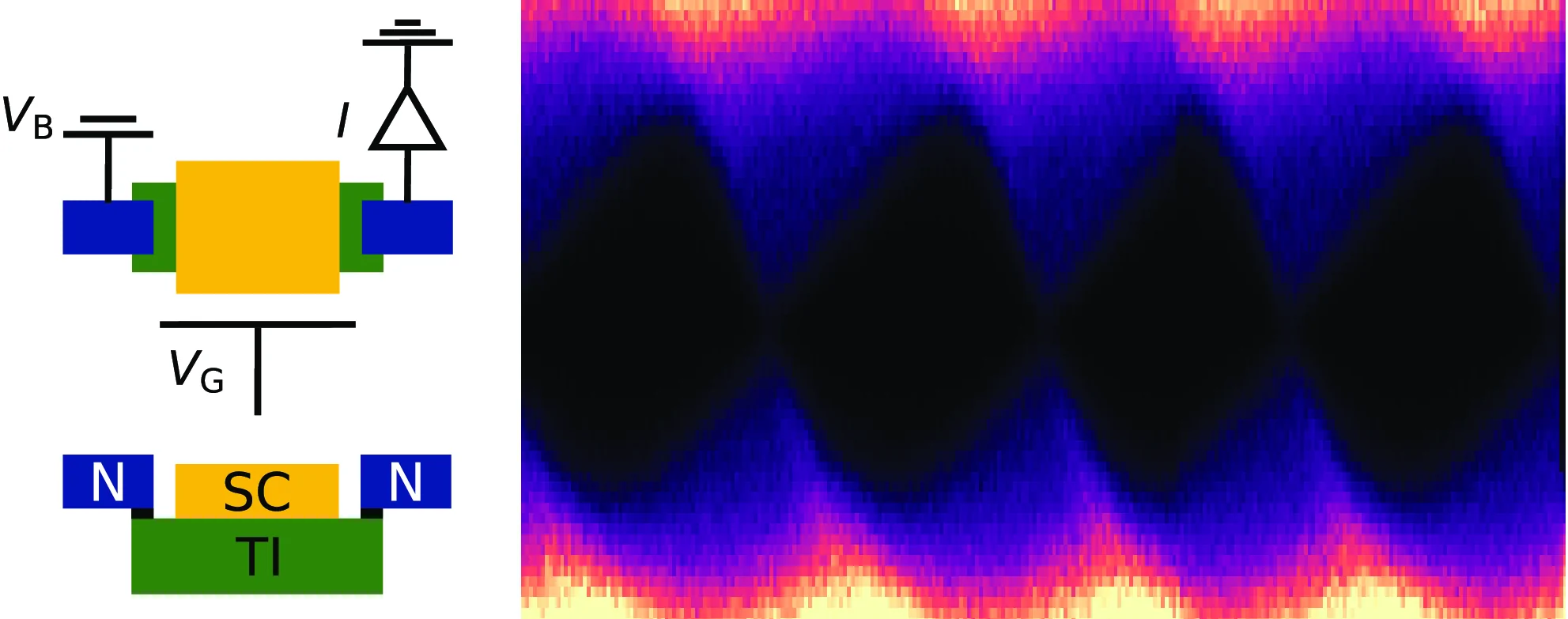

Creating topological superconductivity remains a central objective in condensed matter physics due to its prospective application in fault-tolerant quantum computing.^1^ The essential theoretical building blocks are Majorana zero modes, predicted to emerge in topological systems proximitized by a conventional s-wave superconductor.^2–5^ Semiconductor nanowires with strong spin–orbit coupling have therefore been studied extensively, with early experiments reporting signatures consistent with Majorana physics in nanowires^6^ and superconducting charge islands.^7–10^ These interpretations, however, remain under active debate.^11^

Topological insulators (TIs) provide an alternative platform in which proximity-induced superconductivity is expected to give rise to topological phases.^12^ A key conceptual advantage of TIs is that the topological regime can be realized periodically over a wide energy range by adjusting the chemical potential, the magnetic flux along the wire, or both.^12–14^ Spectroscopy on quasi-one-dimensional TI nanowires offers a controlled setting, as their topological properties are governed by the magnetic flux threading the wire. In particular, for a wire with a uniform cross section, at half-integer flux quanta, Φ = (*n* + 1/2)Φ_0_, the surface-state spectrum is expected to re-enter a topologically non-trivial regime (Figure 1a), while trivial phases occur at integer flux quanta.^14^ This alternating sequence provides a robust experimental fingerprint. Despite these favorable properties, spectroscopic studies of proximitized TIs remain comparatively scarce,^15–18^ with tunnel spectroscopy only recently showing significant progress.^19,20^

**Figure 1:**
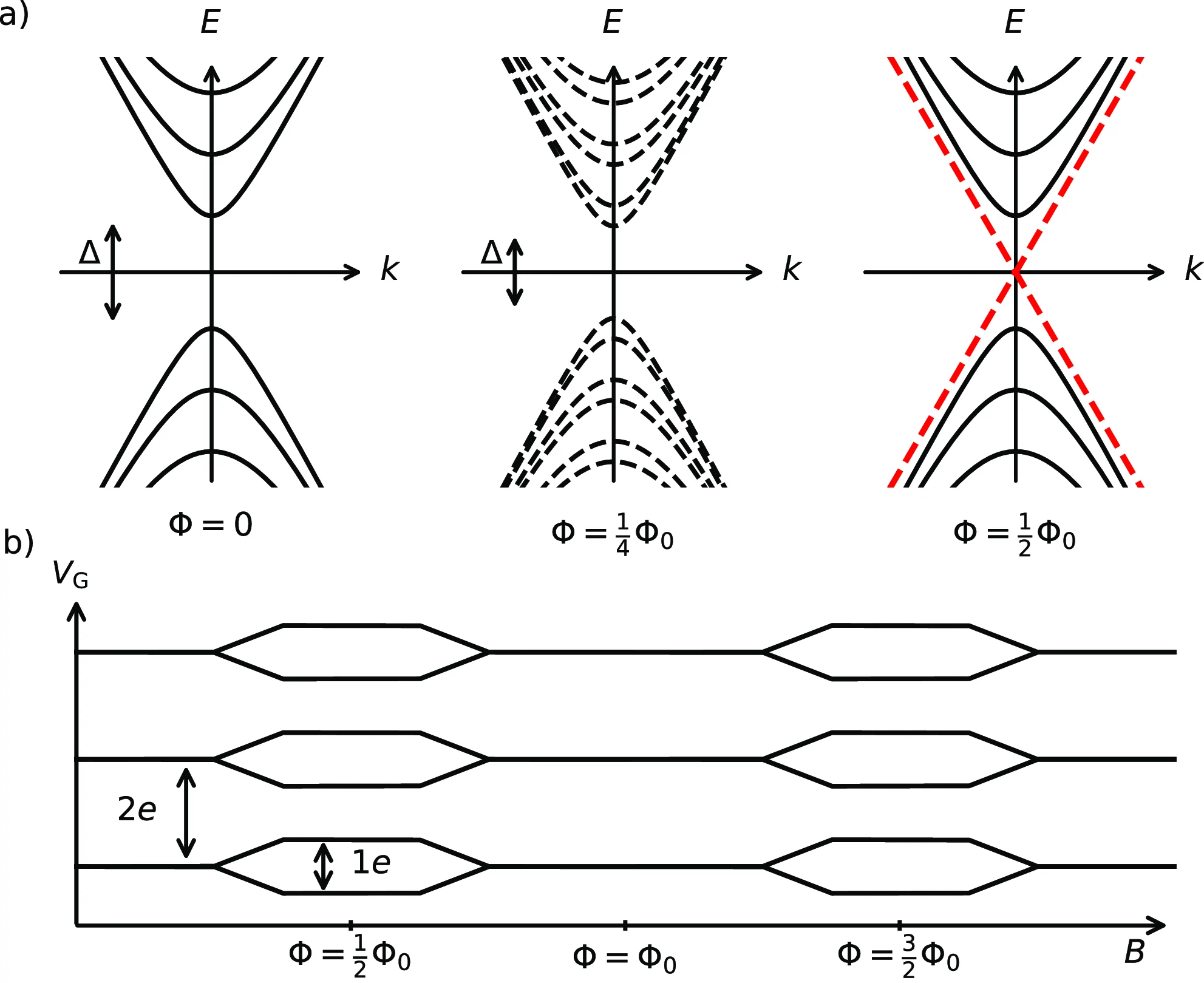
(a) Schematic band structure of a quasi-1D TI nanoribbon as a function of the axial magnetic flux, with a bandgap of Δ. At Φ = (*n* + 1/2)­Φ_0_, the surface-state spectrum (red) re-enters a topological regime. Solid lines indicate doubly degenerate bands, while dashed lines indicate non-degenerate bands. (b) Schematic of Coulomb peaks in a proximitized topological-insulator charge island with periodically appearing zero-bias states. When the topology is restored by flux going through the wire [Φ = (*n* + 1/2)­Φ_0_], Majorana zero modes are predicted to appear and thus lift the 2*e* periodicity. The size of the topological regime depends on the exact position of the Fermi level in the TI.

Charge island hybrid devices constitute a complementary approach to tunneling spectroscopy, as they allow access to charging effects and parity-dependent transport.^8^ Beyond spectroscopy, control over fermion parity is a key requirement for the operation and readout of Majorana-based qubits.^21–24^ While hybrid charge island devices are well-established in semiconductor–superconductor hybrids, their realization in TIs has been limited to a few non-proximitized examples.^25–28^ In a superconducting charge island, the charging periodicity is governed by the competition between the charging energy and the energy of the lowest-lying odd-parity excitation. If odd-parity states remain energetically inaccessible, transport occurs through Cooper pairs and a 2*e* charging periodicity is expected. In contrast, low-energy subgap states reduce the energetic cost of odd-parity charge states and can lead to even–odd or 1*e*-periodic transport.^9^ In a topological regime, a zero-energy state is expected to provide such a low-energy odd-parity state, resulting in a flux-dependent modification of the charging periodicity, as schematically illustrated in Figure 1b. To the best of our knowledge, a proximitized TI charge island has not been demonstrated so far. This absence is largely attributable to two material challenges. First, unlike semiconductors, TIs cannot be fully depleted into a bulk band gap by electrostatic gating due to the presence of metallic surface states. Second, rapid surface oxidation^29^ degrades interface transparency, complicating the formation of a high-transparency superconductor–TI interface.

In this work, we report transport measurements on a proximitized TI charge island fabricated using an advanced *in situ* multi-angle growth technique that directly addresses these challenges. The process enables the formation of pristine interfaces for proximity-induced superconductivity and the realization of well-defined tunneling barriers. We observe robust Coulomb blockade over a wide gate voltage range and a pronounced suppression of low-energy conductance consistent with an induced superconducting gap in the island.

The fabrication process builds on selective area growth (SAG)^30^ combined with shadow evaporation, extending the approach introduced in ref 31. A detailed description of the mask fabrication is provided in the Supporting Information. A lower mask defines the selective growth region for the TI, while an upper, partially free-standing mask enables shadowed deposition of multiple materials. With the control of the evaporation angles and sample rotation, spatial offsets between different layers are achieved without any post-growth processing, allowing the realization of hybrid nanostructures with integrated tunneling electrodes in a single growth sequence.

Figure 2 illustrates the growth procedure. Figure 2a shows a false-color scanning electron micrograph of a representative device, highlighting the TI nanoribbon (green), superconducting island (yellow), normal electrodes (blue), and mask/substrate (gray). The unusually large central opening was chosen to improve imaging of the underlying structure. The double-mask stack consists of a lower 5 nm SiO_2_ and 20 nm Si_3_N_4_ layer defining the trenches for SAG and an upper 300 nm SiO_2_ and 100 nm Si_3_N_4_ layer forming the shadow mask (see Figure 2b). The trenches are patterned by lithography in Si_3_N_4_, followed by wet etching of SiO_2_ to create the partially free-standing geometry.

**Figure 2:**
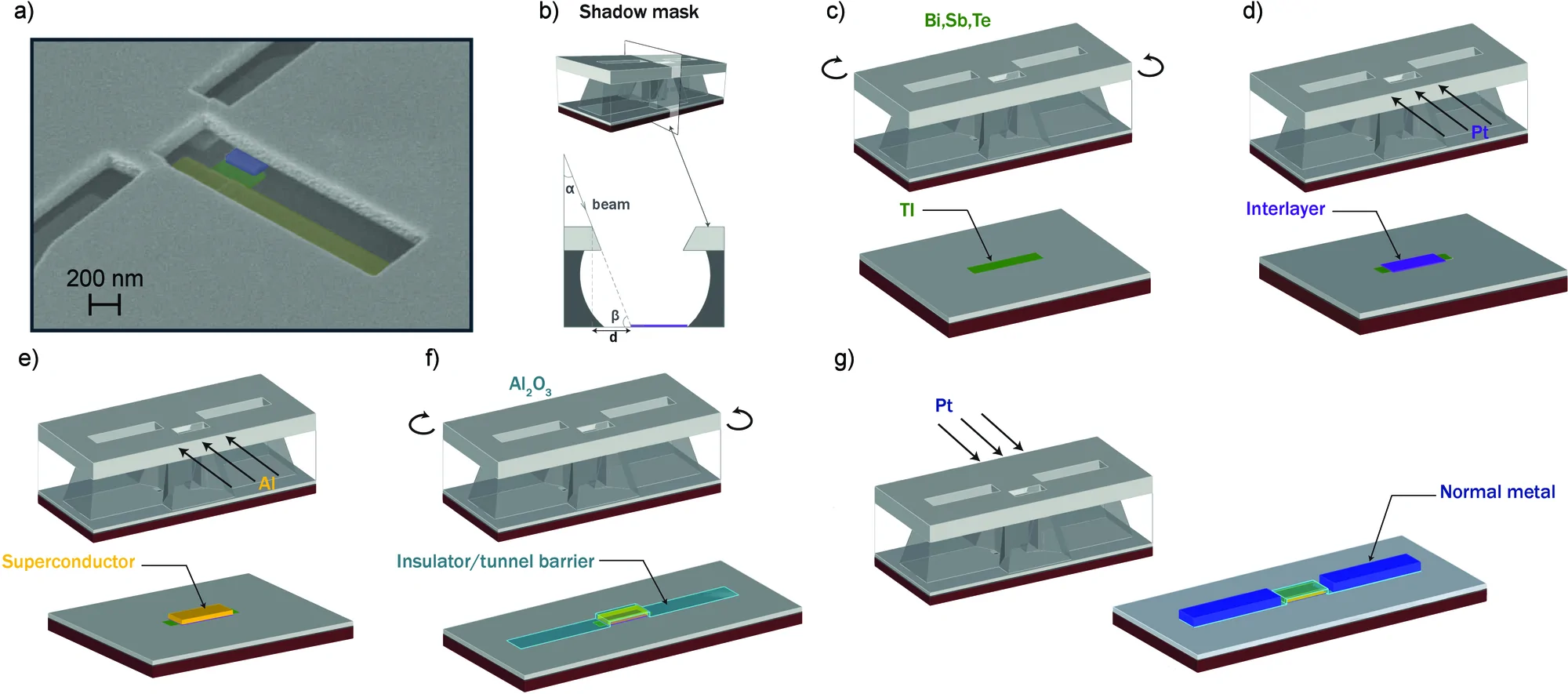
(a) False-color SEM image of a representative device under a tilt of 50° showing the TI ribbon (green), superconducting island (yellow), normal contacts (blue), and mask. (b) Pre-patterned shadow mask with its cross section, showing the partially free-standing mask. (c–g) Schematic of the multi-angle *in situ* fabrication process. The TI is grown under rotation in panel c. A Pt diffusion barrier and Al superconductor are deposited from the same angle without rotation in panels d and e. An Al_2_O_3_ tunnel barrier is grown under rotation in panel f, followed by deposition of normal Pt contacts from the opposite direction in panel g.

Following the recipe in ref 31, the TI nanoribbon is grown first under rotation, confined to the rectangular selective area, to a thickness of 18 nm, width of 200 nm, and length of 1 μm with a composition of Bi_2–*x*_Sb_*x*_Te_3_, with *x* = 1.68 ± 0.04 (see Figure 2c). To suppress interdiffusion between the TI and the superconductor, a 3 nm Pt diffusion barrier is deposited,^32^ followed by a 20 nm Al layer acting as the proximitizing superconductor (Figure 2d and e). Both are deposited from the same direction without rotation and defined by the middle hole in the mask. An oscillatory motion of the substrate holder relative to the material source during deposition can be used to adjust the shadow and increase coverage.^33^ A stoichiometric Al_2_O_3_ tunnel barrier is subsequently grown under rotation to cap the exposed ends of the TI ribbon (Figure 2f). Although the nominal thickness is 5 nm, sequential shadowing by the upper mask during rotation reduces the effective barrier thickness to approximately 1 nm. Finally, 40 nm thick Pt normal electrodes are evaporated from the opposite angle with respect to the superconductor deposition, shown in Figure 2g, and the entire structure is capped with Al_2_O_3_ under rotation to protect the final device from degradation at ambient conditions (not shown).

This fully *in situ* process offers several advantages. It prevents oxidation of the TI surface prior to superconductor deposition,^34^ avoids damage and contamination associated with post-growth etching,^31^ and enables fabrication of many devices on the same chip. Moreover, despite the exponential dependence of tunnel rates on barrier thickness, the resulting barriers are reproducible across devices grown in the same run, enabling more complex multi-barrier architectures.

Electronic transport measurements are performed in a dilution refrigerator with a base temperature of 10 mK and an estimated electron temperature of 50 mK. Standard low-frequency lock-in techniques are employed to measure the differential conductance with an excitation voltage of 7 μV for the data presented in Figures 3 and 4a and b, while 10 μV was used everywhere else including the Supporting Information. A schematic of the measurement configuration is shown in Figure 3a. A voltage bias is applied to one normal electrode, while the other electrode is grounded, and the resulting current is measured. A global back gate is realized by applying a voltage to the metallic back plate of the chip carrier, with the chip made of high-resistivity Si constituting the required dielectric.

**Figure 3:**
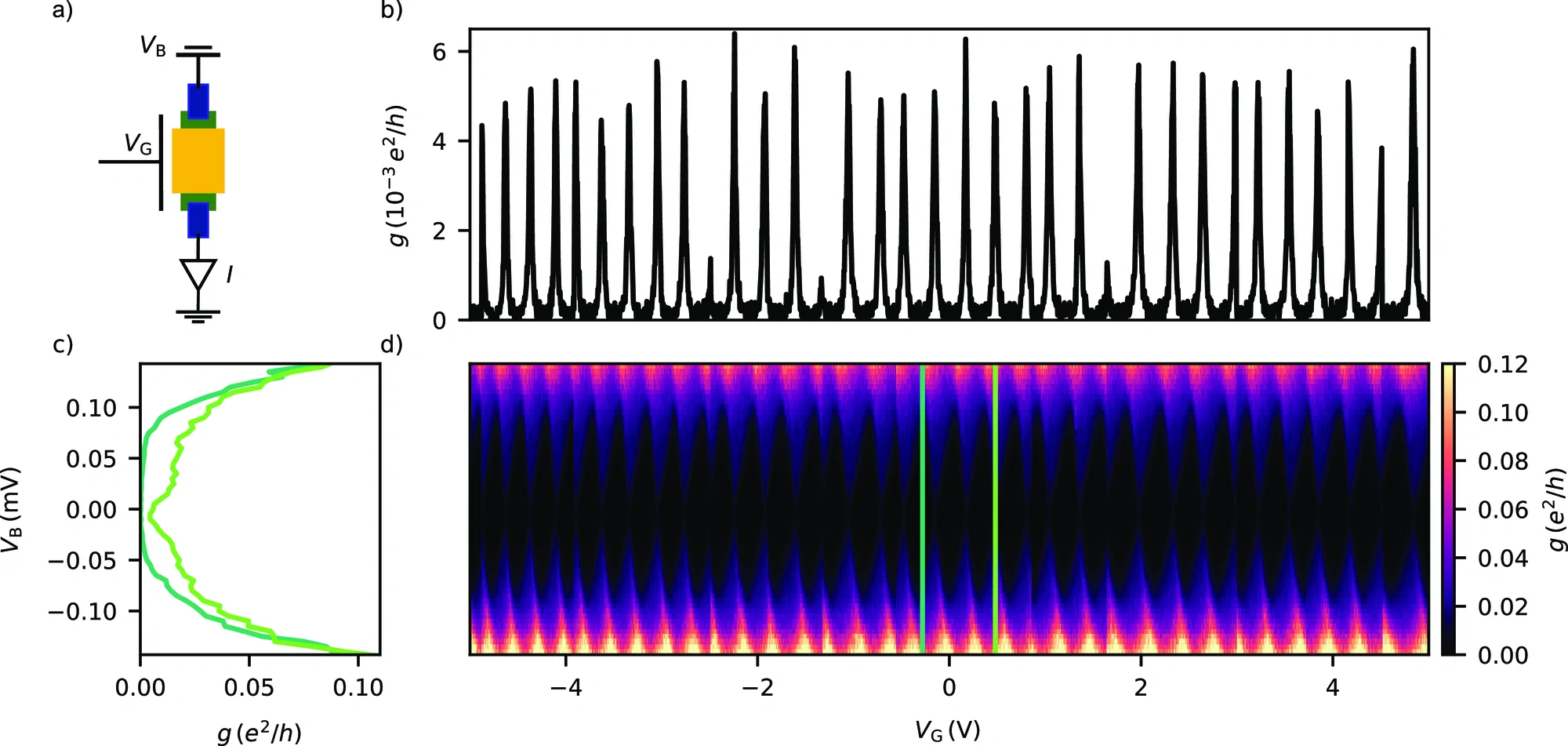
(a) Measurement schematic of the proximitized charge island. A bias voltage is applied to one contact, while the current is measured on the other one. (b) Differential conductance *g* as a function of back-gate voltage at zero bias, showing periodic Coulomb blockade peaks. (c) Differential conductance versus bias voltage, for two line cuts at different gate voltages. In turquoise is a line cut through complete blockade at low bias, while in green, the Coulomb diamond is exactly closed. (d) Coulomb diamonds measured as a function of bias and gate voltage, demonstrating a well-defined charging regime. Line cuts from panel c are marked in the same colors.

**Figure 4:**
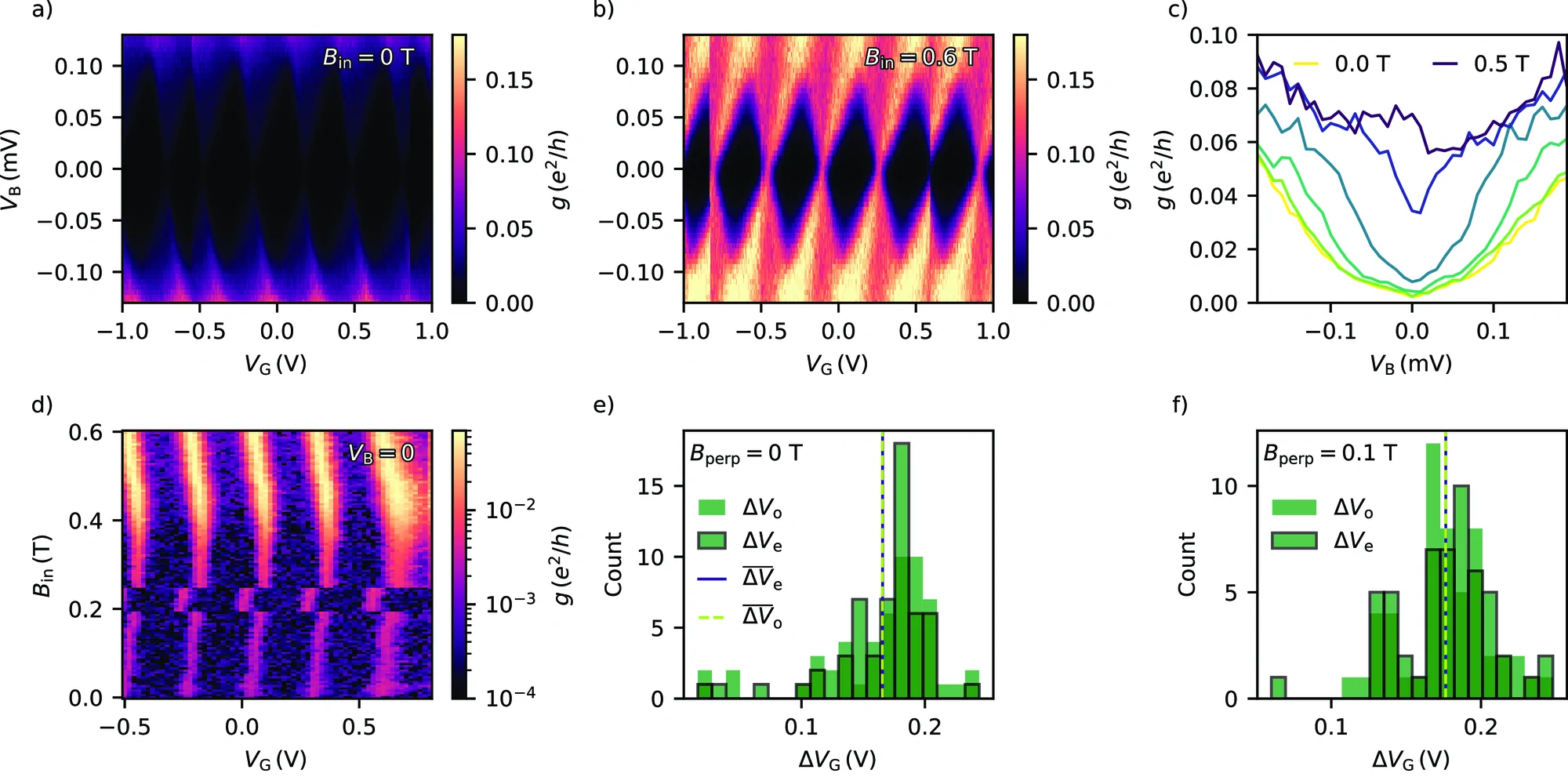
(a and b) Coulomb diamonds at zero in-plane magnetic field and 0.6 T. A pronounced suppression of conductance outside the diamonds is visible at zero field, which gets lifted at high field. (c) Line cuts through the closing points of the Coulomb diamonds at magnetic fields spaced by 0.1 T, highlighting the magnetic-field-dependent conductance suppression associated with proximity-induced superconductivity. (d) Gate voltage versus magnetic field at zero bias. The gate periodicity stays constant, but the conductance increases once the superconductivity is diminished. (e and f) Histograms of even−odd Δ*V*_e_ and odd−even Δ*V*_o_ peak spacings at zero field and high out-of-plane field. The average spacing is marked by a solid blue and dashed green line.

Figure 3b shows periodic conductance peaks as a function of gate voltage at zero bias, characteristic of Coulomb blockade. Bias spectroscopy at a fixed gate voltage (Figure 3c) reveals suppressed conductance at −0.05 < *V*_B_ < 0.08 mV. The two line cuts are taken at *V*_G_ = −0.28 and 0.48 V for turquoise and green, respectively. Combining sweeps of both bias and gate voltage yields well-defined Coulomb diamonds over a wide gate range (Figure 3d), unambiguously demonstrating quantized charge transport through the island. The line cuts in Figure 3c are chosen such that they capture a blockaded configuration (turquoise) and an open one (green). The slight asymmetry of the conductance suppression around zero bias in Figure 3c is attributed to the biasing scheme and asymmetry of the electrical contacts.

From the diamond size, we extract a charging energy of 95 μeV and a gate lever arm of approximately 4 × 10^–4^. The charging energy remains constant over a wide gate range, indicating a highly occupied regime. The extracted charging energy is lower than expected from electrostatic simulations (≈360 μeV), likely due to enhanced screening by the surrounding metallic mask structure, which increases the total island capacitance. Notably, a smaller effective island would yield a larger charging energy; the observed reduction therefore indicates that the island extends across the full TI trench and superconducting island rather than being confined to a subset of it.

To probe the influence of the superconducting proximity effect, Figure 4a and b show Coulomb diamonds at zero field and an in-plane field along the TI wire *B*_in_ = 0.6 T. At zero field, the differential conductance outside the blockade regime is strongly suppressed at low bias, consistent with a proximity-induced gap in the density of states. At high field, where superconductivity is suppressed, this additional reduction in conductance disappears. Figure 4c presents line cuts (positions marked in Figure S2) taken between two Coulomb diamonds, directly illustrating the closing of the induced gap.

Independent evidence for the formation of an induced superconducting gap is obtained from tunnel spectroscopy of a longer proximitized TI nanoribbon fabricated on the same chip and therefore under the same growth conditions (Figure S4). In this device, the superconducting contact is grounded and spectroscopy at one end of the nanoribbon reveals an induced gap of approximately Δ* ≈ 200 μeV. While the magnitude of the induced gap in this extended device does not directly determine the gap of the charge island, the measurement demonstrates that the same fabrication process and growth conditions produce proximity-induced superconductivity in the TI nanoribbon.

In Figure 4d, we map the conductance as a function of the gate voltage and magnetic field at near-zero bias. While the Coulomb peak conductance increases once superconductivity is suppressed (*B*_c,in_ ≈ 0.4 T), the peak spacing remains unchanged across the entire field range. No transition from 1*e* to 2*e* periodicity is observed. The sudden jump near *B*_in_ = 0.2 T is due to charge rearrangements.

Statistical analysis of 108 consecutive Coulomb peaks is shown in Figure 4e and f, where we compare odd-to-even Δ*V*_o_ and even-to-odd Δ*V*_e_ spacings at zero field and a high out-of-plane field (*B*_perp_ = 0.1 T), at which superconductivity in the device is suppressed. Within experimental uncertainty, no statistically significant difference between the average of the two spacings Δ*V̅*_o_ and Δ*V̅*_e_ is found. We also do not find an indication that the distribution of the spacings changes in a systematic manner when the superconductivity is suppressed. The broad distribution of spacings is attributed to charge rearrangements in the electrostatic environment, likely dominated by trapped charges in the surrounding oxides.

The absence of 2*e* periodicity is consistent with the presence of a soft gap in the proximitized island. Importantly, the relevant energy scale for the island parity is the energy *E*_0_ of the lowest-lying odd-parity subgap excitation rather than the magnitude of the superconducting gap Δ* itself.^9^ If sufficiently low-energy subgap states are present, such that *E*_0_ is small compared to the charging energy, odd-parity charge states can become energetically accessible and single-electron transport can occur even in the presence of a finite superconducting gap. In our device, not all electronic states are necessarily proximitized equally strongly, and weakly or non-proximitized states may therefore remain at low energies and prevent 2*e* periodicity. Contributions from the diffusion barrier cannot be excluded and motivate further optimization of material combinations and interface engineering. In addition, the use of normal-conducting leads may further suppress 2*e* periodicity in the island. The leads can be a source of quasiparticles, which are known to prevent parity preservation.^35^ We deliberately avoided superconducting leads in order to unambiguously attribute the observed superconductivity to the proximity effect of the island.

In conclusion, we demonstrate a proximitized topological insulator charge island realized using a fully *in situ* multi-angle fabrication technique. The approach yields clean superconductor–TI interfaces, reproducible tunnel barriers, and scalable device fabrication. Low-temperature transport measurements reveal stable Coulomb blockade and a clear proximity-induced superconducting gap, establishing a new experimental platform for hybrid TI–superconductor systems. While no charge-parity preservation is observed, the results identify key material challenges and provide a foundation for future studies. The fabrication scheme is readily adaptable to tunnel spectroscopy of extended TI nanowires and more complex device architectures. Overall, this work advances experimental control in TI–superconductor hybrids and supports a more systematic exploration of topological superconductivity and Majorana physics in 3D topological insulators.

## Supplementary Material



## Notes

Electrostatic modeling and material parameters, gate-lever-arm extraction, device fabrication and stencil-mask preparation, cross-sectional TEM images and elemental composition maps, independent characterization of proximity-induced superconductivity in a TI nanoribbon, and additional magnetic-field-dependent Coulomb-diamond measurements and line cuts (PDF)
