# Supplementary material for: Proximitized Topological Insulator Charge Island Fabricated via In Situ Multi-Angle Stencil Lithography
Source: Nano Lett. 2026 Oct 5;26(40):13753–8. doi: 10.1021/acs.nanolett.6c02350 (PMC13674338; doi:10.1021/acs.nanolett.6c02350)
Supplement: Supplementary file 1 [file nl6c02350_si_001.pdf]

Supporting Information: Proximitized Topological Insulator Charge Island Fabricated via *In Situ* Multi-Angle Stencil Lithography

Benedikt Frohn[1], Tobias Schmitt[1], Vanessa Serrano[1], Anne Schmidt[1], Michael Schleenvoigt[1], Albert Hertel[1], Benjamin Bennemann[1], Abdur Rehman Jalil[1], Detlev Grützmacher[1], and Peter Schüffelgen*[1]

[1]PGI-9, Forschungszentrum Jülich and JARA Jülich-Aachen Research Alliance, 52425 Jülich, Germany

*Email: p.schueffelgen@fz-juelich.de

Electrostatic properties

The charging energy of the superconducting island was evaluated using a three-dimensional electrostatic model implemented in COMSOL Multiphysics. The simulated structure consisted of a topological-insulator (BST) nanowire contacted by superconducting metallic islands and leads. The BST channel was modeled with a length of $1\,\mu$m and a width of 200 nm. The superconducting island had a length of 800 nm and a width of 250 nm, while the contact electrodes were modeled with a width of 300 nm. To reduce computational complexity, the lithographic mask used during fabrication was omitted from the simulation geometry. This simplification is expected to underestimate the total capacitance and, consequently, overestimate the charging energy relative to the fabricated device. All material parameters were defined for the few-kelvin regime. Superconducting aluminum was represented by a conductivity of $1 \times 10^9\,\mathrm{S\,m^{-1}}$ to approximate ideal metallic behavior within the electrostatic model. Intrinsic silicon was treated as an insulating substrate with a conductivity of $1 \times 10^{-12}\,\mathrm{S\,m^{-1}}$, consistent with carrier freeze-out at cryogenic temperatures. The material parameters used in the simulations are summarized in Table S1.

Material	ε_r	ρ ($\Omega\,$m)	σ ($\mathrm{S\,m^{-1}}$)
Vacuum	1	–	0
Si	11.7	–	1×10^{-12}
Al	1	–	1×10^{9}
Pt	1	2.6×10^{-11}	–
Al_2O_3	9.7	–	–

Table S1: Material parameters used in the COMSOL electrostatic simulations. Values for Pt correspond to cryogenic transport properties in the few-kelvin regime. The conductivity of Al was chosen to approximate superconducting behavior, while the conductivity of intrinsic Si represents the insulating limit arising from carrier freeze-out at low temperature.

The gate lever arm, α_G, quantifies the coupling between the gate voltage and the electrochemical potential of the island. It is extracted from the slopes of the Coulomb-diamond edges. The two slopes, m_1 and m_2, are defined using the convention $m_i = \mathrm{d}V_\mathrm{G}/\mathrm{d}V_\mathrm{B}$. For the asymmetric biasing configuration used here, the inverse gate lever arm is given by the absolute difference between the two slopes,

$$|\Delta m| = |m_1 - m_2| = \frac{1}{\alpha_\mathrm{G}}. \tag{1}$$

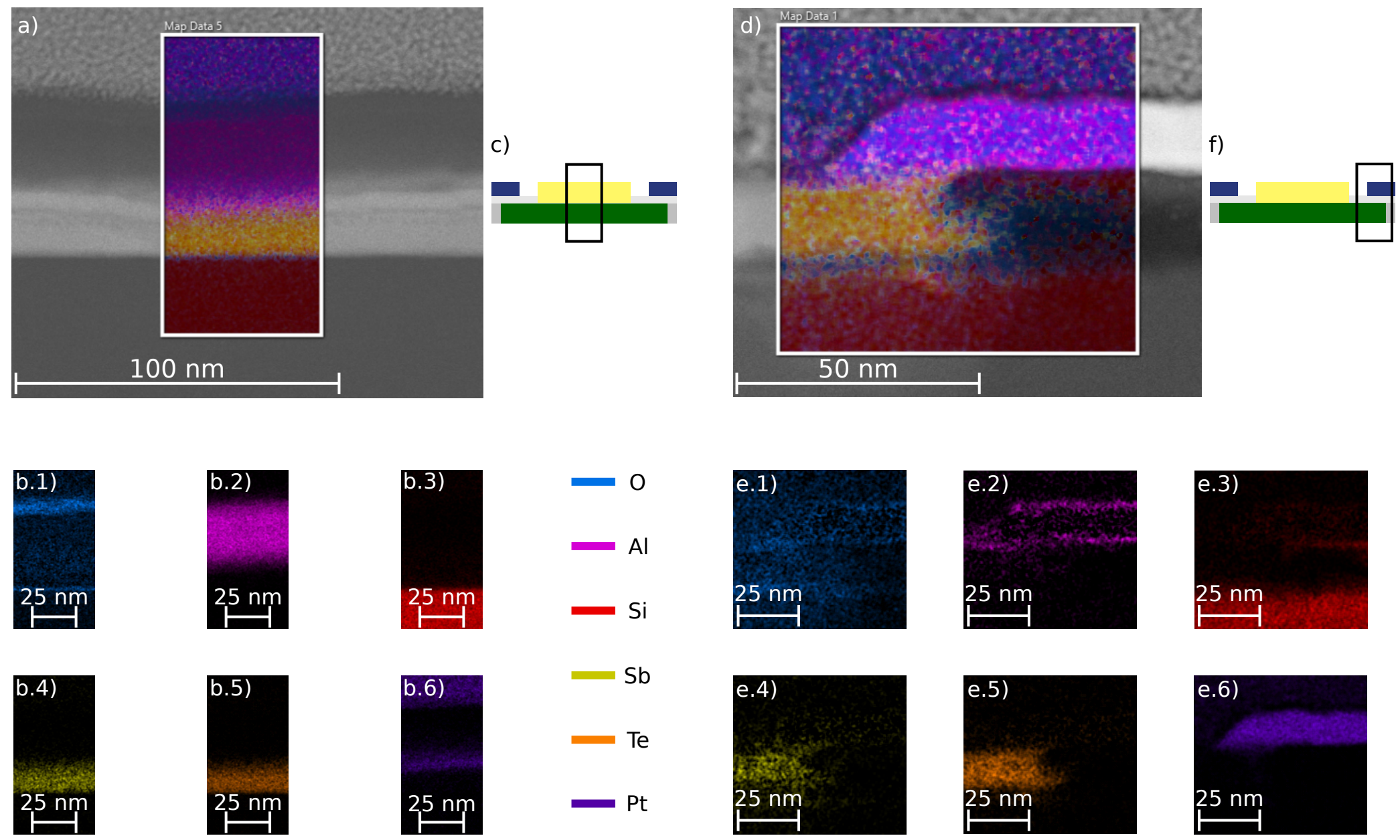

Figure S1: Transmission electron microscopy (TEM) images and elemental composition maps of a device fabricated using the same process as the device studied in transport measurements. Two cross-sectional positions are shown: (a) the Al–Pt–TI interface and (d) the Pt–Al_2O_3–TI interface. Their locations within the device are indicated schematically in (c) and (f), respectively. Panels (b.1–b.6) and (e.1–e.6) show the element-specific signals for the corresponding regions.

Device fabrication and stencil-mask preparation

The devices were fabricated on high-resistivity Si(111) substrates purchased from CrysTec GmbH, with a room-temperature resistivity of 5000–10000 Ω cm. Although the substrates are nominally weakly conducting at room temperature, they become effectively insulating at cryogenic temperatures. The substrate type is equivalent to that used in Ref. [1], although the substrates may originate from a different manufacturing batch.

The double-mask structure was fabricated in two sequential steps. First, the lower selective-area-growth mask was prepared following the process described in Ref. [2]. This mask consists of a thin SiO_2/Si_3N_4 stack with a total thickness of approximately 25–30 nm. Openings in this mask define the exposed Si(111) trenches in which the quasi-one-dimensional topological-insulator nanostructures are grown.

Second, an upper stencil/shadow mask was fabricated on top of the selective-area-growth mask following the approach introduced in Ref. [1]. The upper mask consists of 300 nm of SiO_2 and 100 nm of Si_3N_4. The vertical separation between the lower growth plane and the upper stencil mask, together with the designed lateral openings, defines the shadow geometry used for the angle-selective deposition of the superconducting and normal-metal layers.

All nanoscale openings were patterned by electron-beam lithography. The Si_3N_4 layers were structured by reactive-ion etching using CHF_3/O_2 chemistry. Immediately before loading the samples into the molecular-beam-epitaxy system, the SiO_2 layers were selectively etched using wet HF. This etching step removes the oxide from the bottom of the selective-area-growth trenches, thereby exposing the Si(111) surface for topological-insulator growth. Simultaneously, it underetches the sacrificial SiO_2 layer of the upper mask and releases the free-standing Si_3N_4 stencil bridges.

After loading into the growth chamber, the samples were annealed at 700 °C for 20 min. This annealing step removes the hydrogen termination produced by the HF treatment and exposes reactive Si dangling bonds. During the initial growth sequence, the surface is exposed to Te, which passivates the dangling bonds and forms a suitable interface for the subsequent van der Waals epitaxy of the BST nanoribbon within the predefined selective-area-growth trenches. Full process development of the lower selective-area-growth mask

and the upper stencil-mask process is provided in Ref. [2] and Ref. [1], respectively.

The resulting material stack is illustrated in Fig. S1 using cross-sectional transmission electron microscopy and elemental composition analysis performed on a device fabricated with the same process as the measured devices. The images show the two relevant interface regions, namely the Al–Pt–TI interface and the Pt–Al_2O_3–TI interface. The corresponding elemental maps confirm the spatial arrangement of the deposited layers and demonstrate the successful formation of the intended heterostructure following selective area growth and angle-dependent metal deposition. As the investigated lamella was extracted from a nominally similar device rather than from the device used for transport measurements, the microscopy results are representative of the fabrication process and material stack.

Additional magnetic field measurements

The magnetic-field dependence of the induced superconducting gap is examined for both in-plane and out-of-plane field orientations in Figs. S2 and S3, respectively. The magnetic field applied in each Coulomb-diamond measurement is indicated in the upper-right corner of the corresponding panel. In both configurations, increasing the magnetic field enhances the conductance outside the Coulomb-blockaded regions, consistent with a progressive suppression of the induced gap. For the in-plane field measurements, the dashed lines in Fig. S2 indicate the positions of the line cuts presented in Fig. 4c of the main text. The corresponding out-of-plane field line cuts are shown together with the Coulomb-diamond measurements in Fig. S3.

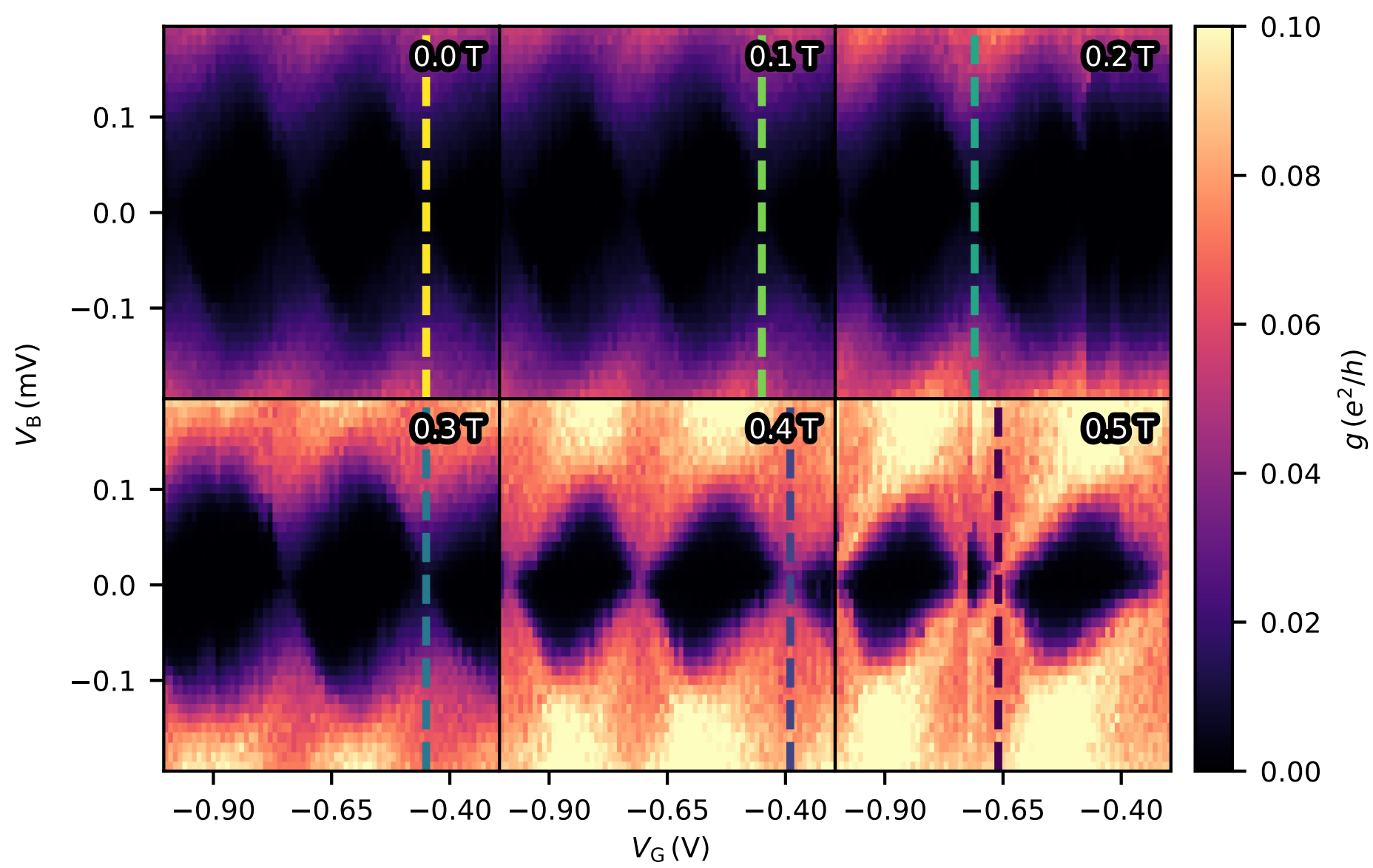

Figure S2: Coulomb-diamond measurements at different in-plane magnetic fields. The magnetic field applied in each measurement is indicated in the upper-right corner of the corresponding panel. With increasing magnetic field, the conductance outside the Coulomb-blockaded regions increases. The dashed lines indicate the positions of the line cuts shown in Fig. 4c of the main text.

Independent characterization of proximity-induced superconductivity

To independently characterize the superconducting proximity effect produced by our fabrication process, we perform tunnel spectroscopy on a longer proximitized TI nanoribbon fabricated on the same chip as the charge-island devices. The device therefore experienced the same TI growth, interface preparation, diffusion-barrier deposition, and superconducting deposition. In contrast to the charge-island geometry, the superconducting electrode is grounded and the differential conductance is measured at one end of the proximitized nanoribbon.

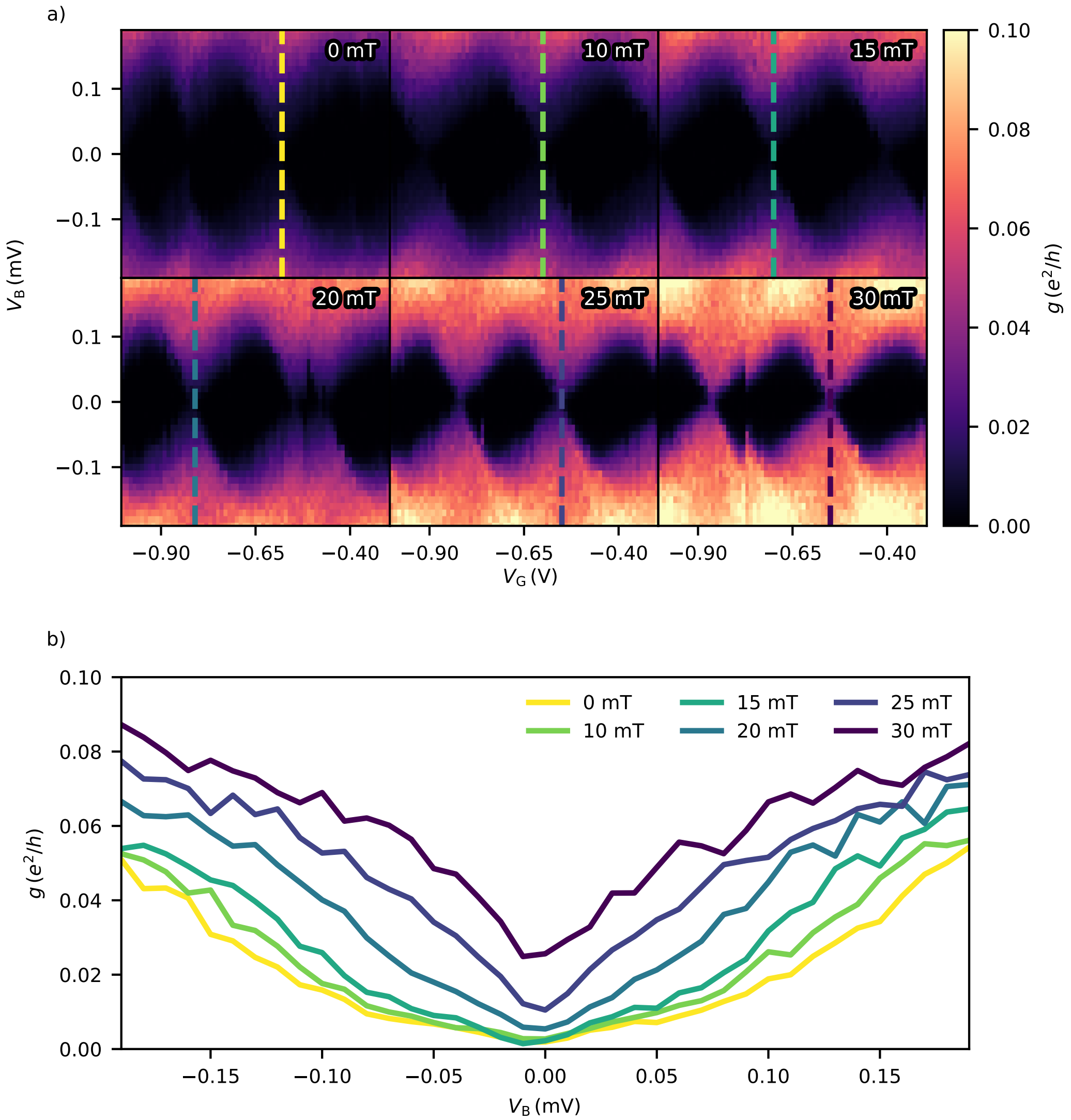

Figure S3: (a) Coulomb-diamond measurements at different out-of-plane magnetic fields. The magnetic field applied in each measurement is indicated in the upper-right corner of the corresponding panel. With increasing magnetic field, the conductance outside the Coulomb-blockaded regions increases. (b) Line cuts at different magnetic fields, taken at the positions marked by the dashed lines in (a).

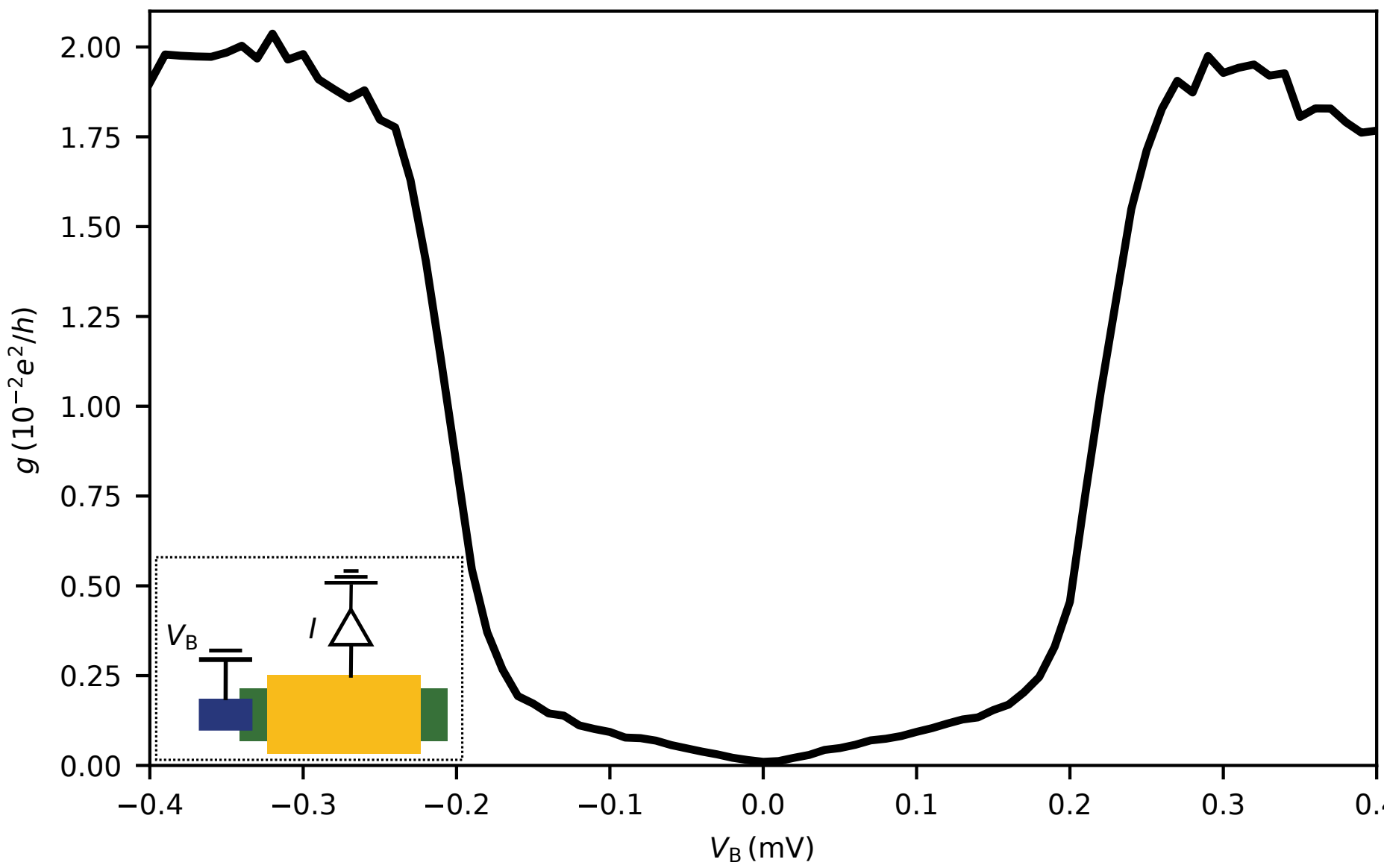

Figure S4: Independent characterization of proximity-induced superconductivity in a TI nanoribbon fabricated on the same chip as the charge-island devices. The differential conductance as a function of bias voltage V_B shows a pronounced suppression around zero bias, corresponding to an induced superconducting gap of approximately $\Delta^* \approx 200\,\mu eV$. Inset: Measurement schematic. The bias voltage V_B is applied to the normal-metal contact (blue) at one end of the TI nanoribbon (green), and the current I is measured through the grounded superconducting electrode (yellow).

Figure S4 shows the differential conductance as a function of bias voltage V_B. A pronounced suppression of conductance is observed around zero bias, corresponding to an induced superconducting gap of approximately $\Delta^* \approx 200\,\mu eV$.

The observation of a well-defined induced gap in this device demonstrates that the fabrication process produces proximity-induced superconductivity in the TI under the same growth conditions as those used for the charge island. The value of Δ^* obtained here should, however, not be interpreted as a quantitative measurement of the induced gap in the charge island, since the strength of the proximity effect can depend on the individual device geometry, interface coupling, and electronic states involved.

References

(1) Schüffelgen, P. et al. Selective Area Growth and Stencil Lithography for in Situ Fabricated Quantum Devices. *Nature Nanotechnology* **2019**, *14*, 825–831.

(2) Jalil, A. R.; Schüffelgen, P.; Valencia, H.; Schleenvoigt, M.; Ringkamp, C.; Mussler, G.; Luysberg, M.; Mayer, J.; Grützmacher, D. Selective Area Epitaxy of Quasi-1-Dimensional Topological Nanostructures and Networks. *Nanomaterials* **2023**, *13*, 354.
